# Exploring Amyloidogenicity of Peptides From Ribosomal S1 Protein to Develop Novel AMPs

**DOI:** 10.3389/fmolb.2021.705069

**Published:** 2021-08-19

**Authors:** Oxana V. Galzitskaya

**Affiliations:** ^1^ Laboratory of Bioinformatics and Proteomics, Institute of Protein Research, Russian Academy of Sciences, Pushchino, Russia; ^2^ Laboratory of the Structure and Function of Muscle Proteins, Institute of Theoretical and Experimental Biophysics, Russian Academy of Sciences, Pushchino, Russia

**Keywords:** amyloid, toxicity, drug, pathogenic organism, aggregation

## Abstract

Antimicrobial peptides (AMPs) and similar compounds are potential candidates for combating antibiotic-resistant bacteria. The hypothesis of directed co-aggregation of the target protein and an amyloidogenic peptide acting as an antimicrobial peptide was successfully tested for peptides synthesized on the basis of ribosomal S1 protein in the bacterial culture of *T. thermophilus*. Co-aggregation of the target protein and amyloidogenic peptide was also tested for the pathogenic ribosomal S1 protein from *P. aeruginosa*. Almost all peptides that we selected as AMPs, prone to aggregation and formation of fibrils, based on the amino acid sequence of ribosomal S1 protein from *E. coli, T. thermophilus, P. aeruginosa,* formed amyloid fibrils. We have demonstrated that amyloidogenic peptides are not only toxic to their target cells, but also some of them have antimicrobial activity. Controlling the aggregation of vital bacterial proteins can become one of the new directions of research and form the basis for the search and development of targeted antibacterial drugs.

## Introduction

Antibiotic resistance of bacteria is a pressing global problem. The rate of development and introduction of new antibiotics for clinical use lags behind the spread of antibiotic resistance (Theuretzbacher et al., 2020). It should be noted that among antimicrobial drugs, only seven peptide antibiotics were approved for use, and of more than 10,000 antimicrobial peptides (AMPs), only 61 are at the stage of preclinical and clinical trials (Koo and Seo, 2019), which demonstrates the complexity of the development and implementation of new antimicrobial substances. One of the possible solutions to combat pathogenic microorganisms is the development and use of new antimicrobial peptides (AMP) (Kurpe et al., 2020; Lee et al., 2020).

The general mechanism of AMPs includes permeating membranes, facilitating membrane remodeling processes such as pore formation and fusion (Lewies et al., 2019), but peptides with alternative mechanisms of action look promising. Working on the amyloidogenic properties of proteins and peptides, O.V. Galzitskaya suggested the possibility of directed co-aggregation of an amyloidogenic peptide and a target protein, which *in vivo* can manifest itself as an antimicrobial effect. Analysis of the literature confirmed this possibility (Kagan et al., 2012; Last and Miranker, 2013; Zhang et al., 2014; Wang et al., 2016; Soundrarajan et al., 2019; Lee et al., 2020; Gaglione et al., 2021). Last and Miranker showed that amyloidogenic peptides as well as antimicrobial peptides can inhibit bacterial cell growth (Last and Miranker, 2013). The facts of the formation of fibrils by antimicrobial peptides and, conversely, the manifestation of antimicrobial activity of amyloidogenic regions of proteins indicate the presence of a certain connection between them (Last and Miranker, 2013; Zhang et al., 2014). Despite the low similarity between AMPs and amyloidogenic peptides, the latter exhibit similar activity, leading to cytotoxic effects (Zhang et al., 2014). It is believed that mature amyloids do not exhibit toxicity (Gosztyla et al., 2018). On the other hand, there is growing evidence that oligomers of amyloidogenic peptides exhibit antimicrobial activity (Kagan et al., 2012). In any case, it remains unclear how antibacterial activity and the ability to form fibrils are related. Understanding the dependence of the properties of a peptide molecule on its structure is an important component for explaining the nature of a particular phenomenon. Artificially synthesized peptides can be an ideal model for studying the relationship between antimicrobial activity and the ability to form fibrils.

## Choice of Target Protein

**FIGURE 1:**
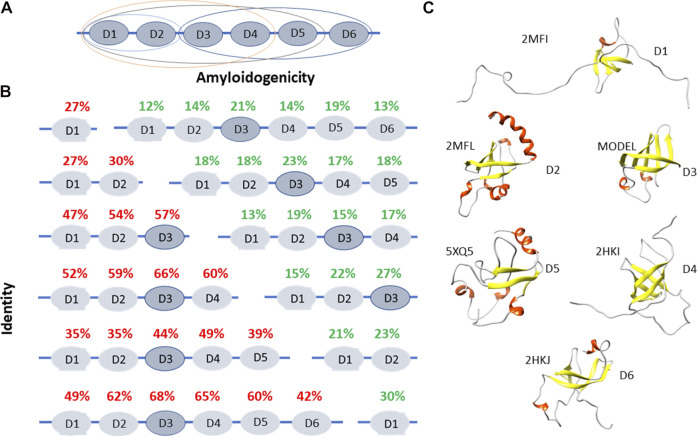
Structural and functional features of ribosomal S1 protein. **(A)** The domain and functional organization of the S1 protein from *E. coli* are marked with different ovals. D1-D2 domains interact with the 30 S ribosomal subunit, D3-D6 domains interact with RNA (Suryanarayana and Subramanian, 1979; Subramanian, 1983). D1-D4 domains interact with the 30 S subunit, are involved in the initiation and translation of synthetic mRNA (poly (U) (Subramanian, 1985). D3 domain is of fundamental importance when interacting with mRNA and tmRNA. It is also important when interacting with ribonuclease regB (McGinness and Sauer, 2004). **(B)** Identity and amyloidogenicity of the 1,453 sequences of ribosomal S1 proteins from 25 different phyla. **(C)** Spatial structures from the Protein Data Bank for D1, D2, D4, D5, and D6 domains of ribosomal S1 protein from *E. coli*. Structure for D3 was predicted using the Robetta server.

We chose the ribosomal S1 protein as a target because it is a unique protein for a bacterial cell. This protein has a number of important functions (participates in translation initiation, translation regulation), its knockout leads to cell death, and is present only in bacteria. We carried out a bioinformatics study of its properties (Deryusheva et al., 2019, 2021; Machulin et al., 2019, 2019). Unique characteristics have been found for this protein. The S1 protein consists of several repeats of the S1 domain (OB-fold), and the number of such repeats depends on the type of bacteria to which this protein belongs. The number of repeats ranges from one to six, and all Gram-negative bacteria have six domains in the ribosomal S1 protein (Machulin et al., 2019). It turned out that this protein is important for the bacterial cell, since mutations in this protein lead to cell death. Since the function of each domain is not fully defined, each amyloidogenic site from different domains will have a different effect. However, the coaggregation of a protein with the peptide will lead to disruption of the functions of that protein, which will be tantamount to protein knockout. Structural and functional features have been well studied so far only for the ribosomal S1 protein from *E. coli* (Figures 1.A,C). It was demonstrated that D1-D2 domains of the ribosomal S1 protein of *E. coli* have high homology (67%) and both are responsible for the interaction with 30 S ribosomal subunit (Suryanarayana and Subramanian, 1979; Subramanian, 1983). Domains D3-D6 interact with RNA (Suryanarayana and Subramanian, 1979; Subramanian, 1983). D3 domain is of fundamental importance in the interaction with mRNA and tmRNA, as well as in the interaction with ribonuclease regB (McGinness and Sauer, 2004). Moreover, D6 domain is an autogenous repressor of its own synthesis (Boni et al., 2000). These facts make the S1 protein an important target for the development of antibacterial drugs (Zhi et al., 2019). The spatial structure was determined for the five domains from the six (except for D3) of the S1 protein from *E. coli* (Figure 1.C).

## Peptides Prone to Aggregation From the Ribosomal S1 Protein

**FIGURE 2:**
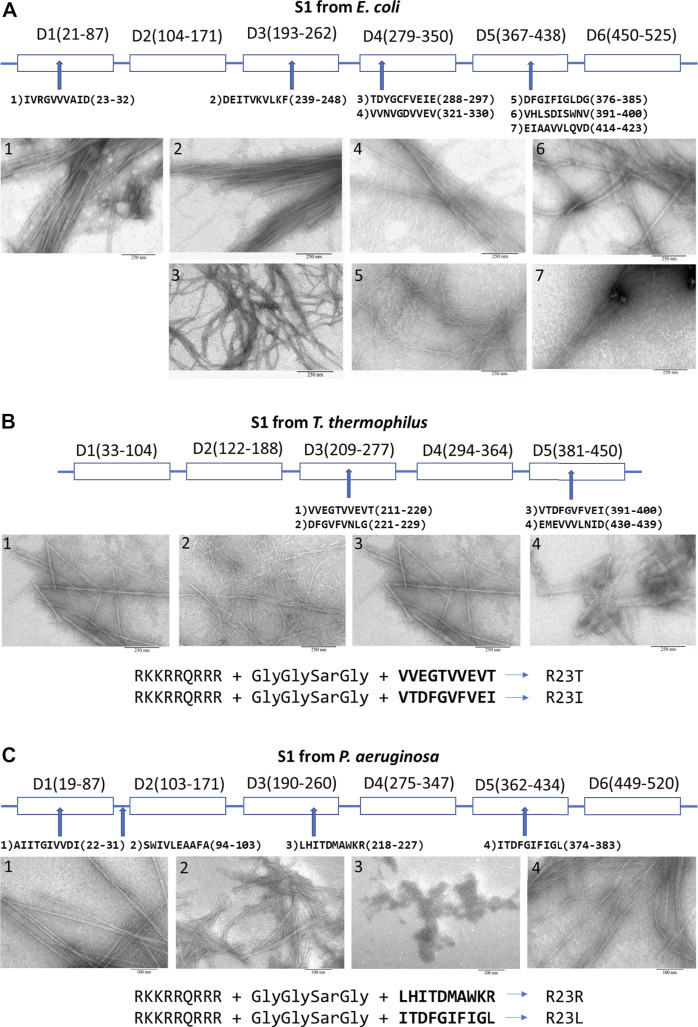
Peptides prone to aggregation from the ribosomal S1 protein. From **(A)** *E. coli*; **(B)** *T. thermophilus* (Grishin et al., 2020b); **(C)** *P. aeruginosa* (Grishin et al., 2021). Scale bar is 250 nm for **(A)**, **(B)** and 100 nm for **(C)**. Amyloidogenic regions were predicted by using four programs: FoldAmyloid, Waltz, PASTA2.0, and Aggrescan (see Supplementary Material).

Domains of the ribosomal S1 protein from *E. coli* (six domains — 557 amino acid residues), *T. thermophilus* (five domains — 536 amino acid residues), and *P. aeruginosa* (six domains — 559 amino acid residues) were analyzed to select regions of the amino acid sequence of the protein potentially possessing amyloidogenic and antimicrobial properties. All these bacteria are Gram-negative. Based on the theoretical analysis using four programs (FoldAmyloid (Garbuzynskiy et al., 2010), Waltz (Maurer-Stroh et al., 2010), PASTA2.0 (Walsh et al., 2014), and Aggrescan (Conchillo-Solé et al., 2007)) to predict amyloidogenic regions, we selected and synthesized seven amyloidogenic peptides from S1 *E. coli*, four peptides from S1 *T. thermophilus,* four peptides from *P. aeruginosa* (see Figure 2 and Supplementary Material). All peptides are 10 amino acid residues long, except for one peptide from S1 *T. thermophilus*. Most of the synthesized peptides, mainly correspond to *β*-strands in the S1 domains of the ribosomal S1 protein (Grishin et al., 2020a).

For membrane penetration, four modified peptides were engineered by adding cell penetrating peptide (CPP) via an additional linker (GlyGlySarGly, where Sar is sarcosine) to the peptide. A fragment of Tat-HIV-1 (49–57) was added to four sequences of the selected peptides to increase an antimicrobial activity, membrane permeability, rigidity and mechanical stability of aggregate complexes (Console et al., 2003; Figure 2). We have verified that this CPP does not have an antibacterial effect on these bacteria.

## Identity and Amyloidogenicity of the S1 Domains in the Ribosomal S1 Proteins

As mentioned above, the ribosomal S1 protein has a limited number of domains from one to six. And, as we mentioned, all Gram-negative bacteria have six repeats of the S1 domains.

Using bioinformatics tools, 1,453 sequences of ribosomal S1 proteins from 25 different phyla were studied (Deryusheva et al., 2019; Machulin et al., 2019, Machulin et al., 2019; Deryusheva et al., 2021). S1 proteins with one domain have a low percentage of identity with each other (27%). With an increase in the number of domains in a protein, the identity of each domain between different representatives of bacteria increases, with the exception of five-domain proteins. The structural scaffold (OB-fold) is more important than the amino acid sequence (Deryusheva et al., 2019; Machulin et al., 2019). This observation confirmed the statement about the uniqueness of each individual domain in the one-domain S1 proteins. The central part of the proteins (third domain) is more conserved than the terminal domains (Figure 1B).

The primary structures of ribosomal S1 proteins contain regions prone to aggregation and formation of amyloids according to four programs: FoldAmyloid (Garbuzynskiy et al., 2010), Waltz (Maurer-Stroh et al., 2010), PASTA2.0 (Walsh et al., 2014), and Aggrescan (Conchillo-Solé et al., 2007). FoldAmyloid prediction data for 1,453 amino acid S1 sequences are shown in Figure 1B. The third domain has the highest identity among various bacterial species, i.e. the most conserved and contains the largest number of amyloidogenic regions. For Gram-negative bacteria containing six domains, the fifth domain is also one of the amyloidogenic domains.

Another interesting question is how often the selected amyloidogenic regions for synthesis are found in other bacterial and eukaryotic proteins. It turns out that we did not find them in eukaryotic proteins, which is very important in order not to initiate directed aggregation with any target protein that includes a similar amino acid sequence.

## Amyloidogenicity of Peptides and Formation of Fibrils

Peptides predicted by bioinformatics tools to aggregate and form amyloid fibrils have been synthesized and tested for their ability to form amyloid fibrils. Despite the strong tendency towards the aggregation of several amyloidogenic sites in the ribosomal S1 protein family, the process of fibril formation is still poorly understood. The S1 proteins studied by us from four organisms (*M. mobili*, *T. thermophilus, P. aeruginosa* and *S. aureus*) did not form amyloid fibrils (Grishin et al., 2020); moreover, the protein from *M. mobile* dropped out into inclusion bodies during isolation (not published data). In this case, all short peptides, except one (we did not deal with a special selection of conditions), formed amyloid fibrils (see Figure 2) (Grishin et al., 2020a; Grishin et al., 2021). The described specific amyloidogenic regions are indeed responsible for the process of fibrillogenesis and can be potential targets for modulating the amyloid properties of bacterial ribosomal S1 proteins.

Coaggregation of peptide and S1 protein was tested for 2 S1 proteins and 5 peptides: S1 *T. thermophilus* with V10T, R23T, R23I (Kurpe et al., 2020; Supplementary Table S1), and S1 *P. aeruginosa* with R23R, and R23L at a ratio of 1:5 (0.5 mg/ml and 2.5 mg/ml) (see Supplementary Table S1). In all these cases, the co-aggregation led to the formation of aggregates of different sizes and fibrils of different diameters. Upon co-aggregation of the protein and peptide, leading to the formation of amyloids, we observed a significant increase in the fluorescence intensity of thioflavin T compared to the S1 protein and the peptide itself (Kurpe et al., 2020; Grishin et al., 2021) (see Supplementary Table S1).

Thus, the ribosomal S1 protein of *E. coli*, *T. thermophilus*, and *P. aeruginosa* contains amyloidogenic sequences that can lead to aggregation of peptide molecules with each other or with other proteins that have aggregation sites (directed coaggregation mechanism). S1-related domains are found in other bacterial proteins, which may increase the number of targets for the peptide.

## Testing for Antibacterial Activity and Toxicity

Peptides predicted by bioinformatics tools as prone to aggregation and formation of amyloid fibrils were synthesized and tested for antimicrobial and cytotoxic effects.

Several peptides have been tested for antimicrobial activity. Peptides from *E. coli* against *E. coli*, and peptides from *T. thermophilus* against *T. thermophilus* cells (Kurpe et al., 2020; Kurpe et al., 2021). Although, as it turned out, it is possible to conduct cross-sectional studies, as our studies have shown for pathogenic cells (results are not published). Certain peptides may indeed have unique properties for different bacterial cultures.

Previously, we were able to evaluate the antimicrobial activity of peptides synthesized on the basis of the amino acid sequence of the ribosomal S1 protein from *T. thermophilus*, which suppressed the growth of *T. thermophilus* cell culture. We have successfully tested this approach on *T. thermophilus* bacterial culture. Among the peptides from S1 *T. thermophilus* studied by us, the most effective peptide was the R23I peptide (minimum inhibitory concentration (MIC) is 50 μg/ml), the effect of which was comparable to that of the antibiotic kanamycin (Kurpe et al., 2020). Another important fact was discovered that CPP decreases amyloidogenicity, but increases antibacterial activity (Kurpe et al., 2020). The antibacterial effect of peptides prone to aggregation and formation of fibrils based on the amino acid sequence of the ribosomal S1 protein from *E. coli* did not show such success as for *T. thermophilus*. Probably, the opportunistic *E. coli* bacterium has mechanisms of resistance to the studied peptides. But, most likely, it is necessary to check our peptides once again, since among the peptides there is the D10G peptide, which is very similar to the peptide V10I from *T. thermophilus* with antibacterial effect. It is necessary to check its effect by adding CPP (Tat-HIV-1 (49–57)), then the peptide will be very similar to R23I. In the case of the pathogenic bacterium *P. aeruginosa*, the antibacterial properties among the peptides synthesized on the basis of the predicted amyloidogenic regions of S1 from *P. aeruginosa* exhibited the R23L peptide, for which the MIC was 8 μg/ml, which is comparable to the action of the antibiotic gentamicin (paper is being prepared). It should be noted that the two antibacterial peptides (V10I from *T. thermophilus* and I10L from *P. aeruginosa,* see Figure 2C) are 63% identical in amino acid sequence, and both belong to the fifth domain.

It should be noted that if amyloidogenic peptides are toxic to cells, they will not be good templates for antibiotic development. Therefore, it is necessary to carry out a test for the survival of eukaryotic cells. In our case, cell viability was estimated by resazurin cell viability assay. Human fibroblast cell survival was 70% for the peptides from *T. thermophilus* (Kurpe et al., 2020) and 100% for the peptides from *P. aeruginosa* (Supplementary Figure S4) in the peptide concentration range of 0.01–20 μg/ml (7.6 µM), which overlaps with the MIC concentration for the R23L peptide.

## Discussion

Currently, there are many programs for predicting the antimicrobial activity of peptides (for example, AMPA or AmPEP (Torrent et al., 2012; Bhadra et al., 2018)). Despite the wide variety of approaches to assessing antibacterial activity, it is difficult to create a universal template that could distinguish between antimicrobial and non-antimicrobial peptides, which is a significant limitation in the development of new AMPs. Obviously, there is a need for laboratory testing of the effectiveness of predicted AMPs, including for further refinement and improvement of the results of the predictive programs (Fields et al., 2020). We have proposed a new mechanism of AMP action, a mechanism of directed co-aggregation, which is based on the interaction of a peptide capable of forming fibrils with a target protein. Amyloidogenic peptides acting on the basis of targeted coaggregation with bacterial ribosomal S1 protein and disrupting its function may be potential antibacterial peptides. Elucidation of the properties of co-aggregates is an important element in the development of antimicrobial peptides acting on the basis of directed co-aggregation. This direction is promising for the opening of new AMPs. The selection of fragments of the amino acid sequence of peptides with potential antimicrobial properties should include other characteristics in addition to the presence of amyloidogenic regions, for example, cell penetrating peptides, which, as we have shown with Tat-HIV-1 (49–57) peptide, for example, weaken the aggregation properties, but enhance the antimicrobial effect of the peptide (Kurpe et al., 2020).

Despite the low ability of bacteria to exhibit resistance to antimicrobial peptides, in order to prevent and resist the emergence of new antibiotic-resistant mutants, it is important to assess the adaptive ability of pathogenic bacteria to peptides. Proteomic profiling of bacteria makes it possible to assess molecular responses in general, which can help in identifying molecular strategies for adapting pathogens to adverse conditions and determining the potential anger of mutant strains.

It can be noted that we were just lucky, having looked at about 20 peptides from three organisms, we were able to find two peptides with antimicrobial properties and low MIC, which is comparable to the MIC of antibiotics. Moreover, AMPs against *P. aeruginosa* showed no toxicity to eukaryotic cells at all.

## Conclusion

We have demonstrated that amyloidogenicity may be one of the important properties of AMPs. We suggested that AMPs form aggregates with target proteins by their amyloidogenic regions, which ultimately lead to cell death. This may indicate that the ability to aggregate may be combined with antimicrobial action against bacteria.

Thus, if we are talking about two sides of the same coin, we can emphasize that, on the one hand, the formation of amyloids can be functional, and on the other hand, it can be associated with a disease, and co-aggregation also has two sides: on the one hand, it is associated with infectivity, on the other hand, direct co-aggregation may be one of the possible mechanisms of AMP action.

## Data Availability

Publicly available datasets were analyzed in this study. This data can be found here: Supplementary Material from PMID: 32707977 and https://www.mdpi.com/1422-0067/22/14/7291/pdf.
