# Supplementary material for: Exploring Amyloidogenicity of Peptides From Ribosomal S1 Protein to Develop Novel AMPs
Source: Front Mol Biosci. 2021 Aug 19;8:705069. doi: 10.3389/fmolb.2021.705069 (PMC8416663; doi:10.3389/fmolb.2021.705069)
Supplement: Supplementary file 1 [file DataSheet1.docx]

Exploring amyloidogenicity of peptides from ribosomal S1 protein to develop novel AMPs

Oxana V. Galzitskaya ^1,2*^

^1^Laboratory of Bioinformatics and Proteomics, Institute of Protein Research, Russian Academy of Sciences, Pushchino, Moscow Region, Russia

^2^Laboratory of the structure and function of muscle proteins, Institute of Theoretical and Experimental Biophysics, Russian Academy of Sciences, Pushchino, Moscow Region, Russia

*** Correspondence:**Dr. Oxana V. Galzitskaya

[ogalzit@vega.protres.ru](mailto:ogalzit@vega.protres.ru)


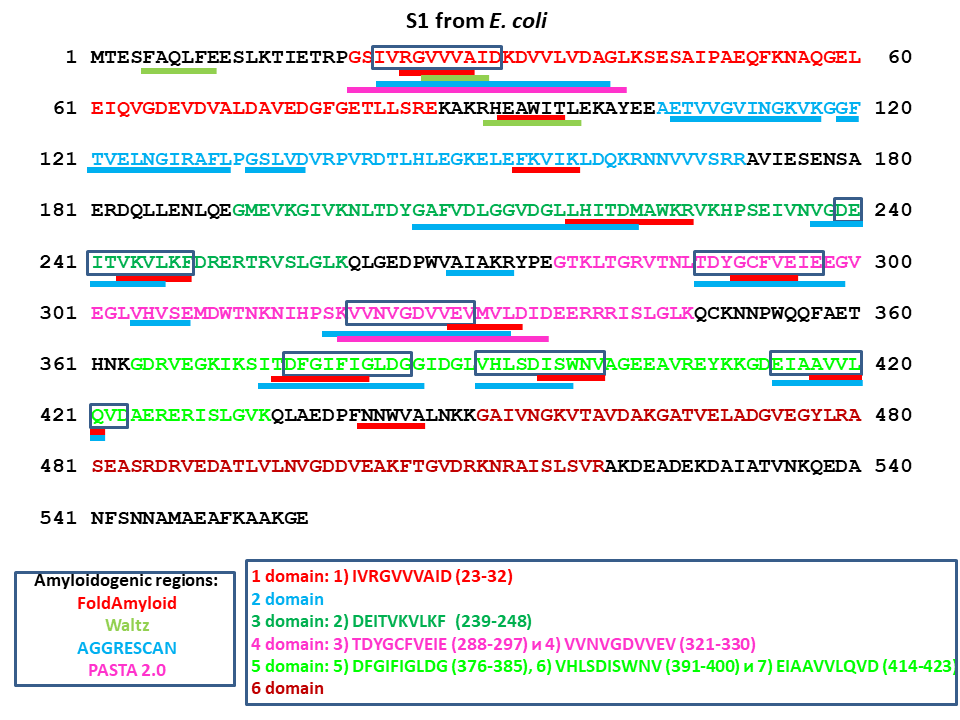


**Figure S1.** Prediction of amyloidogenic regions prone to aggregation. Amyloidogenic fragments for synthesis are outlined in the rectangles.


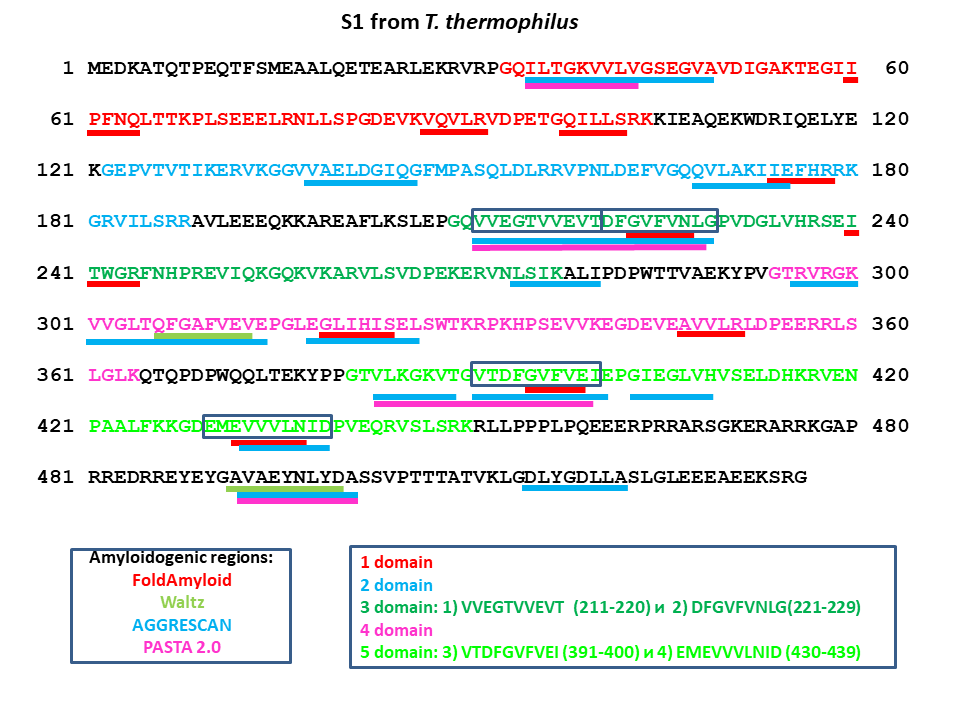


**Figure S2.** Prediction of amyloidogenic regions prone to aggregation. Amyloidogenic fragments for synthesis are outlined in the rectangles.


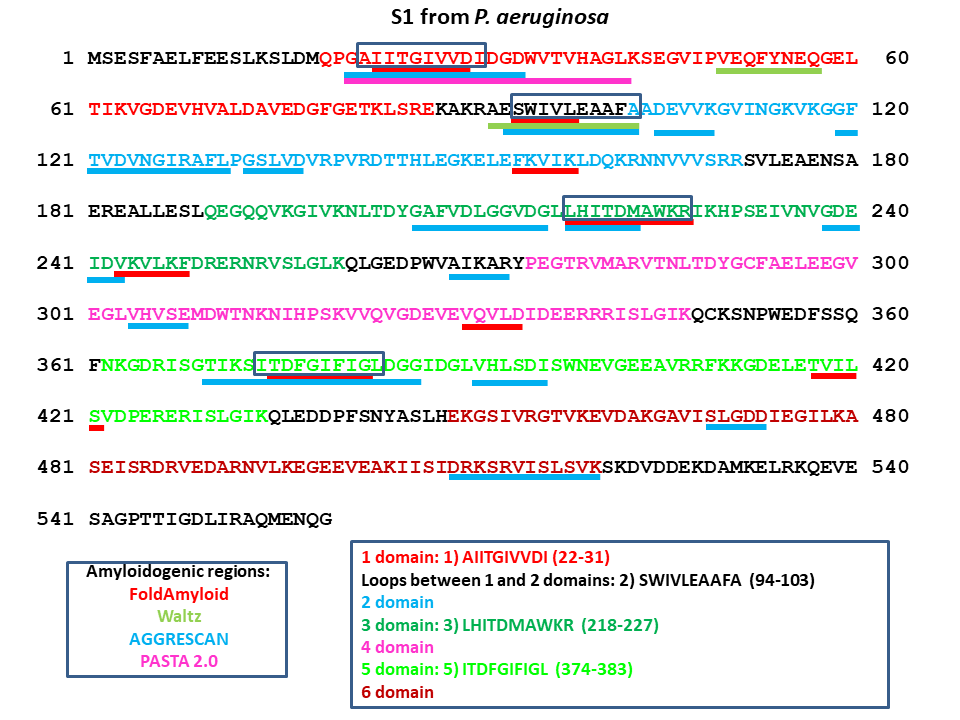


**Figure S3.** Prediction of amyloidogenic regions prone to aggregation. Amyloidogenic fragments for synthesis are outlined in the rectangles.


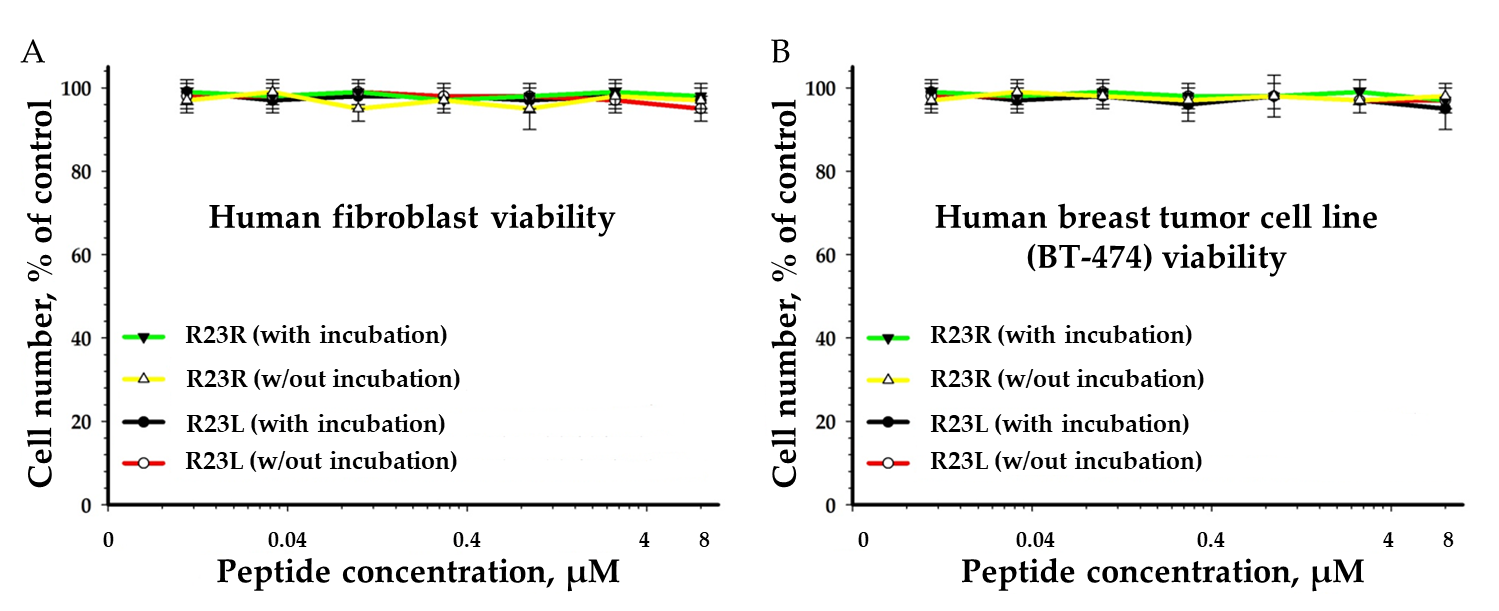


**Figure S4.** Effects of peptide treatment on survival the human fibroblasts (A) and breast tumor cell line BT-474 (B). Error bars show standard errors.

**Supplementary Table 1.** Results of experiments on studying the kinetics of preparations of possible coaggregation for amyloidogenic peptides based on protein S1 and the whole model protein S1 from T. thermophilus and P. aeruginosa under conditions of 50 mM TrisHCl, pH 7.5; 150 mM NaCl.

| 1 |  | S1 from *T. thermophilus* (0.5 mg/ml)  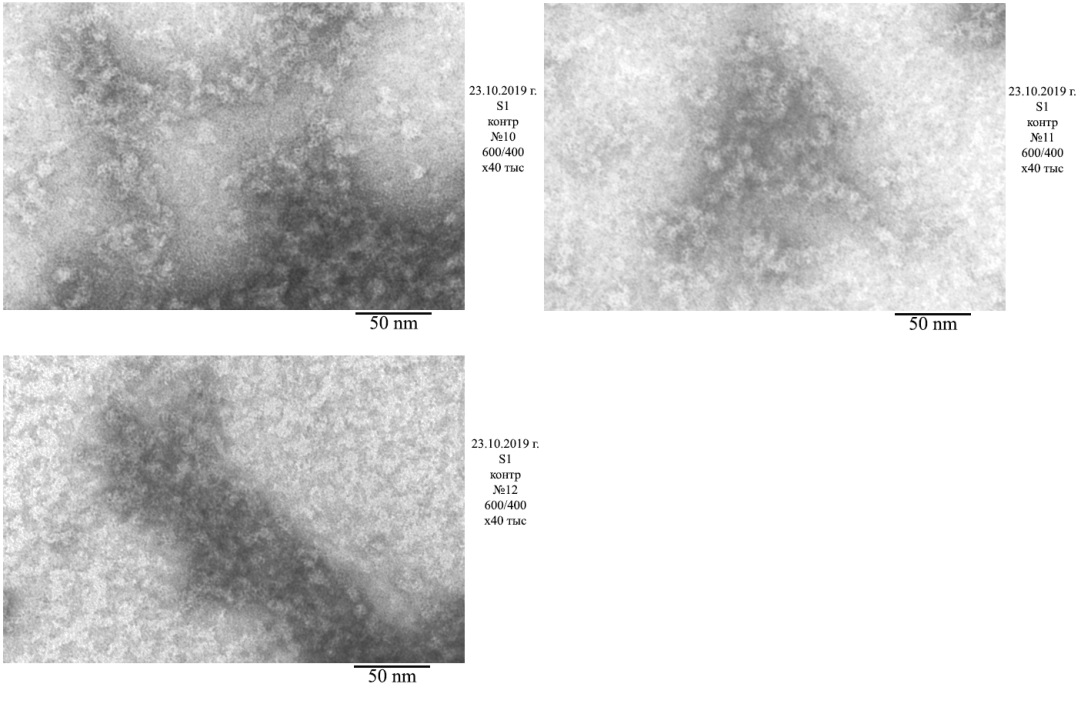 | According to the data of fluorescence spectroscopy, upon joint incubation of the protein and the V10T peptide, the fluorescence intensity increases (from 6 to 6 units at a ratio of 1: 1 and to 120 units at a ratio of 1: 5), which indicates the formation of amyloids.  The V10T peptide and the S1 protein form aggregates. At the same time, it is noticeable that these aggregates are morphologically slightly different - the components of the aggregates of the V10T peptide are somewhat smaller than those of the S1 protein.  When the V10T peptide and S1 protein are mixed in a 1:1 ratio, only aggregates are observed.  When the V10T peptide and S1 protein are mixed in a 5:1 ratio, both aggregates and fibrils are observed. |
| --- | --- | --- | --- |
|  |  | V10T from *T. thermophilus* (0.5 mg/ml)  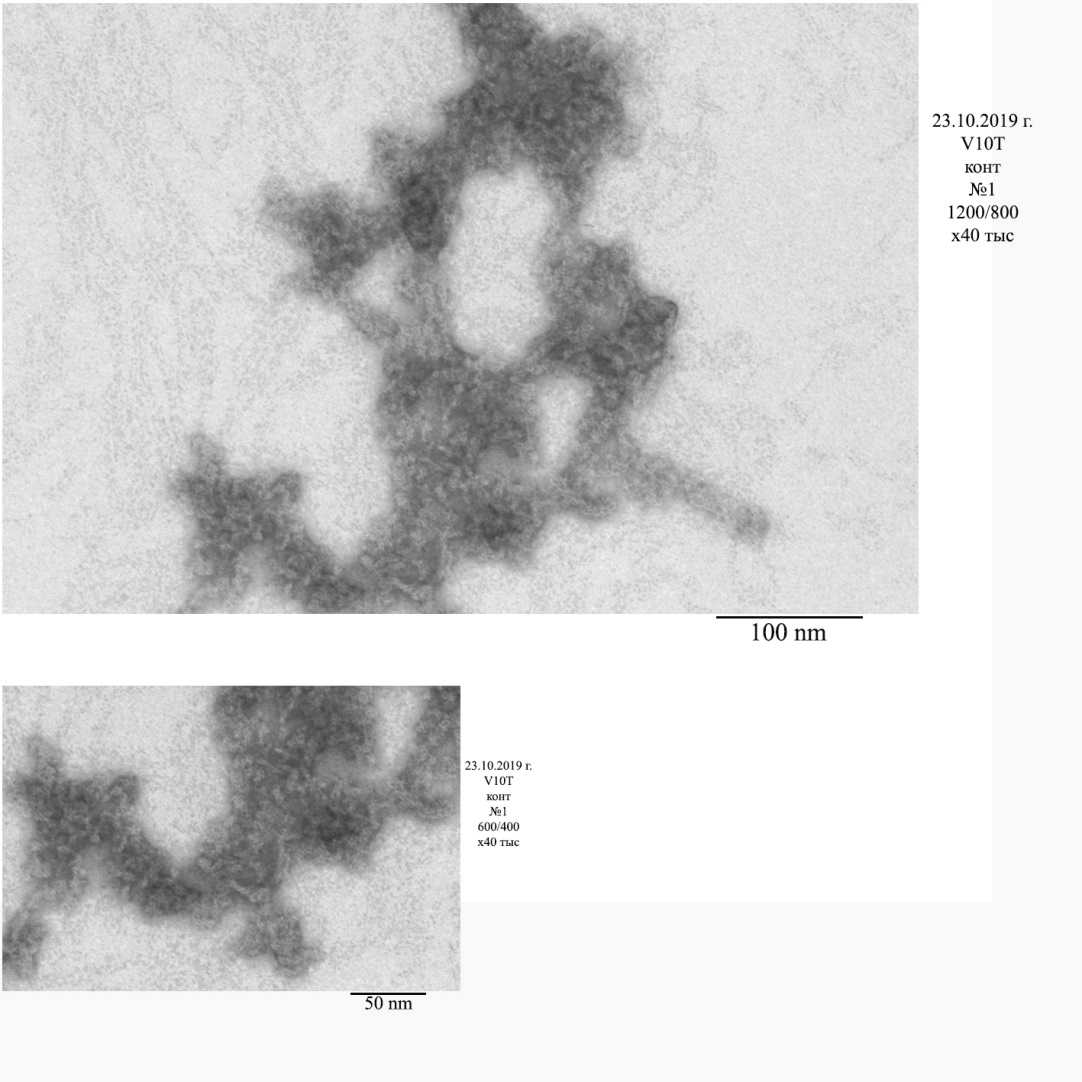 |  |
|  |  | S1:V10T=1:1 (0.5 mg/ml) from *T. thermophilus*  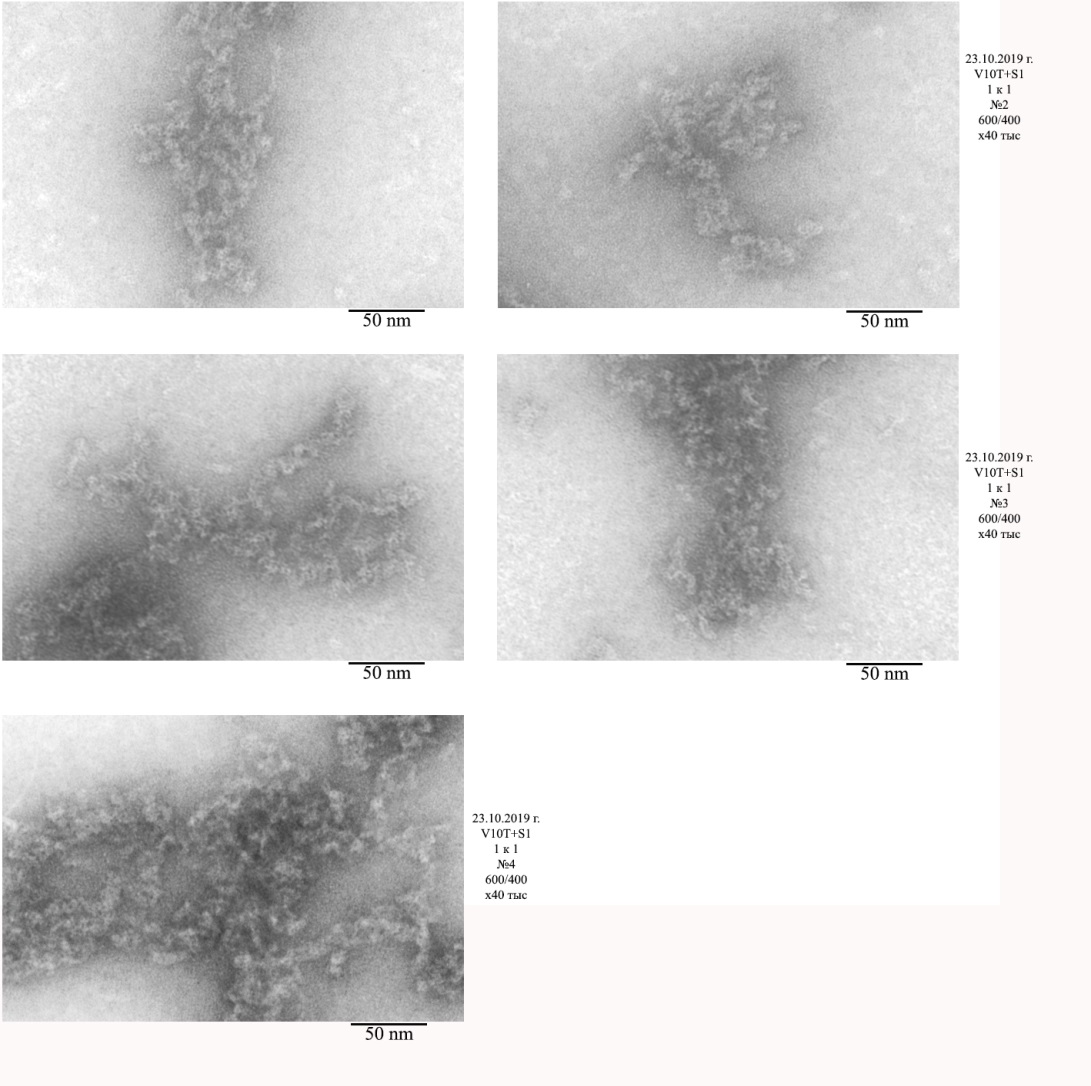 |  |
|  |  | S1:V10T=1:5 (0.5 mg/ml and 2.5 mg/ml) from *T. thermophilus*  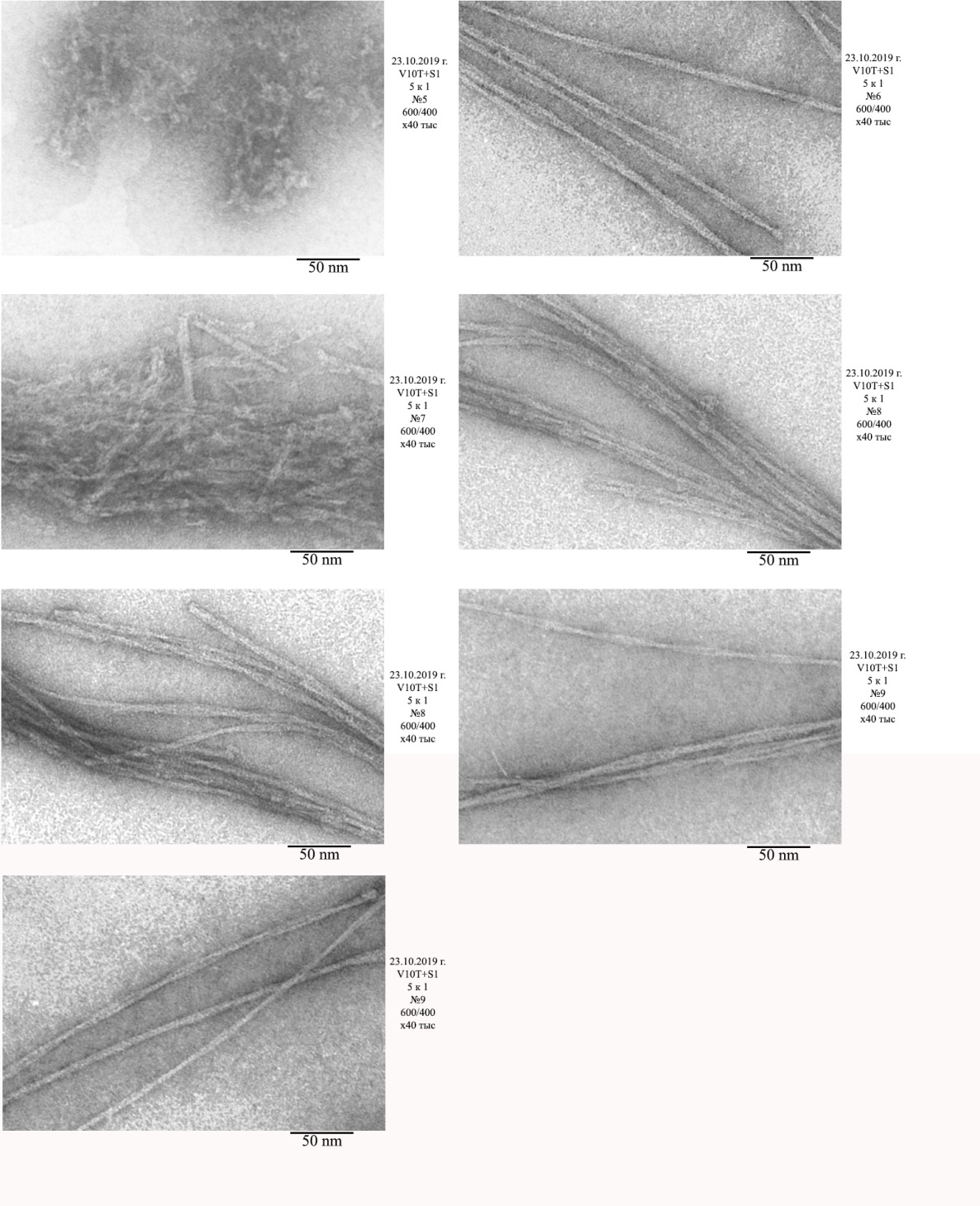 |  |
| 2 |    | S1 (0.5 mg/ml) from *T. thermophilus*  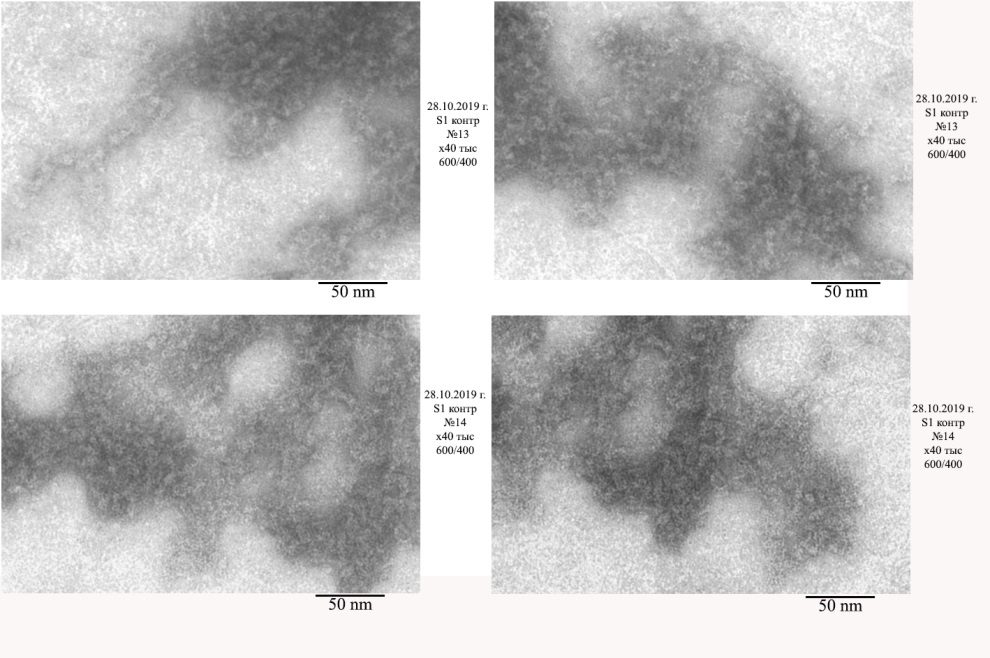 | According to the data of fluorescence spectroscopy, upon joint incubation of protein and peptide, the fluorescence intensity increases insignificantly (from 2 to 3 units at a ratio of 1:1 and to 4.5 units at a ratio of 1:5), which indicates the absence of formation of amyloids or their insignificant amount (at ratio S1: R23I = 1:5).  During co-incubation of the R23I peptide with S1 protein from *T. thermophilus* in a 5:1 ratio, both protein aggregates and fibrils are observed. The number of fibrils increases, and their morphology is similar to that of fibrils when the ratio of peptide and protein is 1:1. |
|  |  | R23I (0.5 mg/ml) from *T. thermophilus*  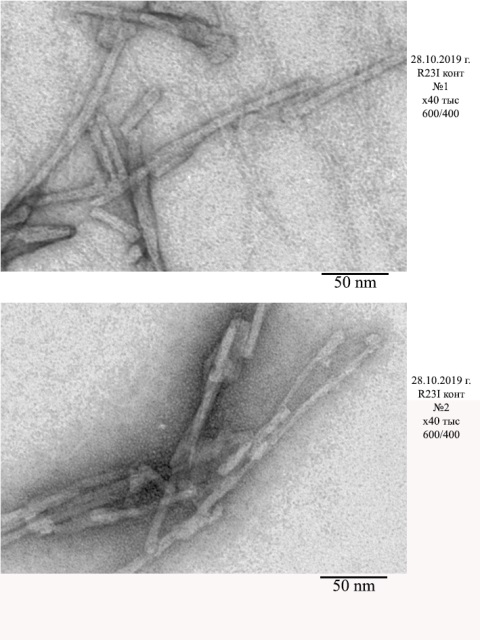 |  |
|  |  | S1:R23I=1:1 (0.5 mg/ml) from *T. thermophilus*  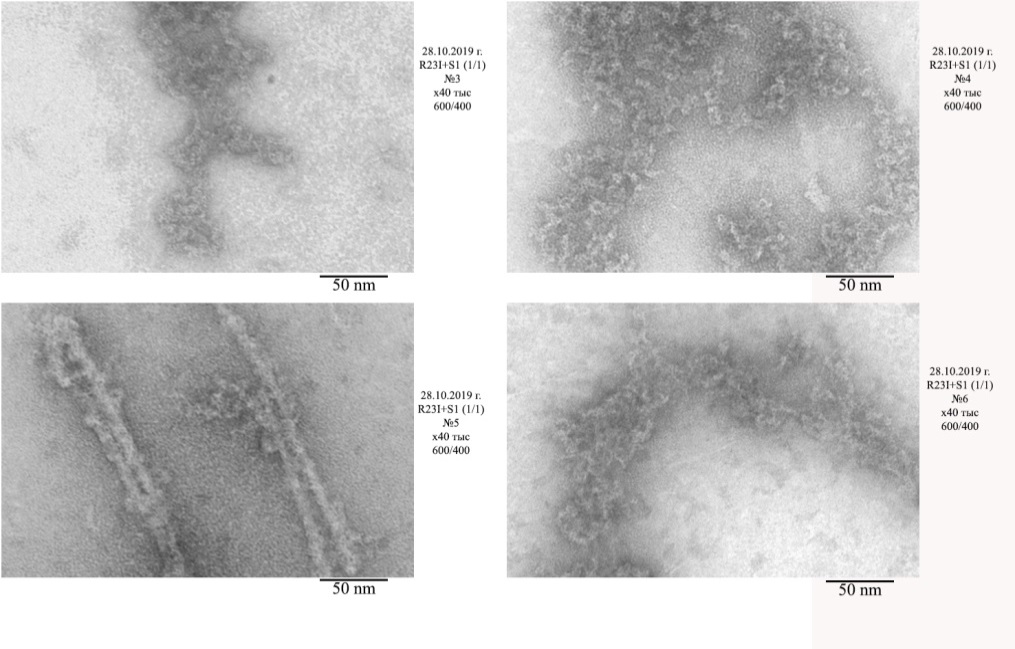 |  |
|  |  | S1:R23I=1:5 (0.5 mg/ml and 2.5 mg/ml) from *T. thermophilus*  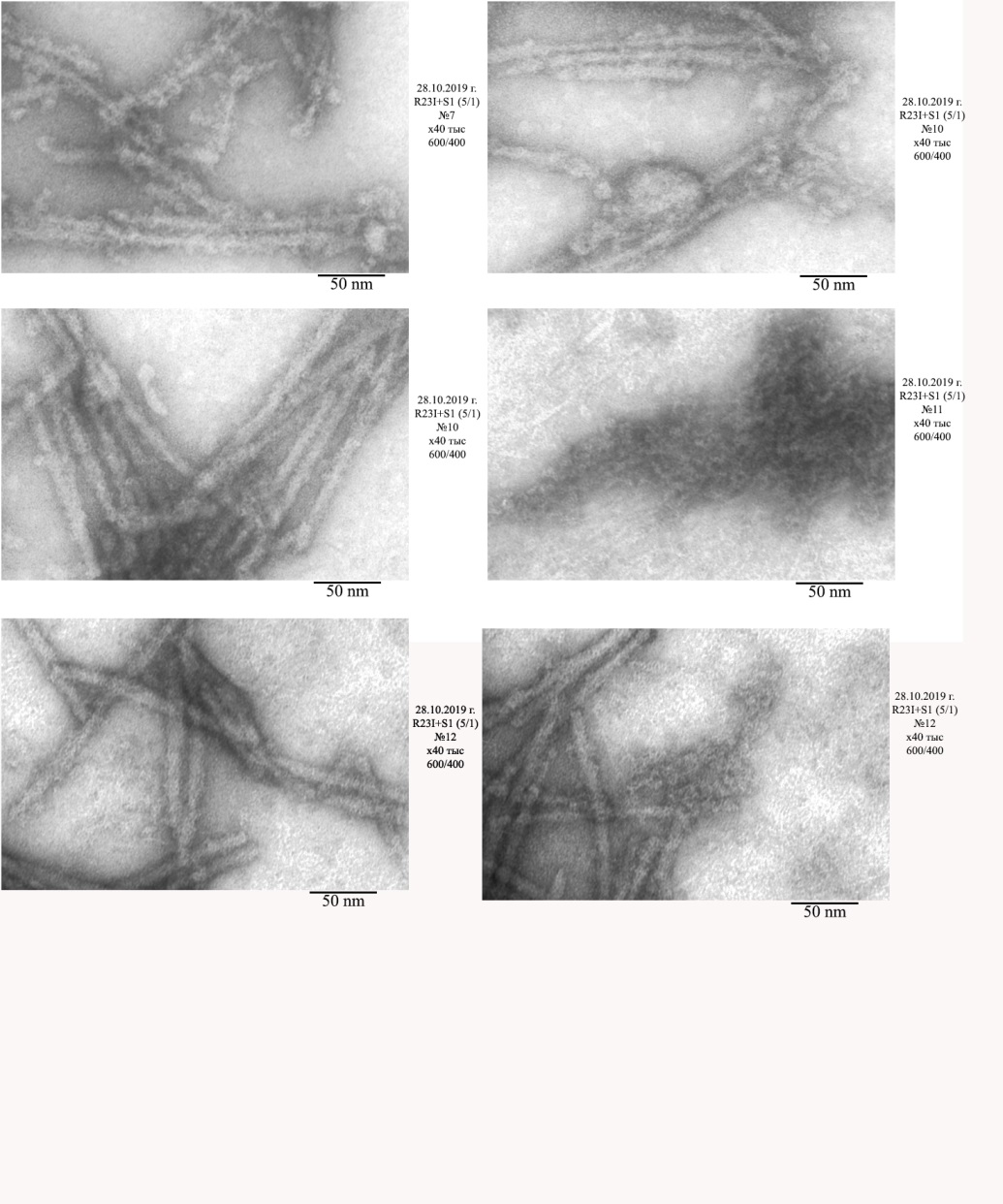 |  |
| 3 |  | S1:R23T=1:5 (0.5 mg/ml and 2.5 mg/ml) from *T. thermophilus*  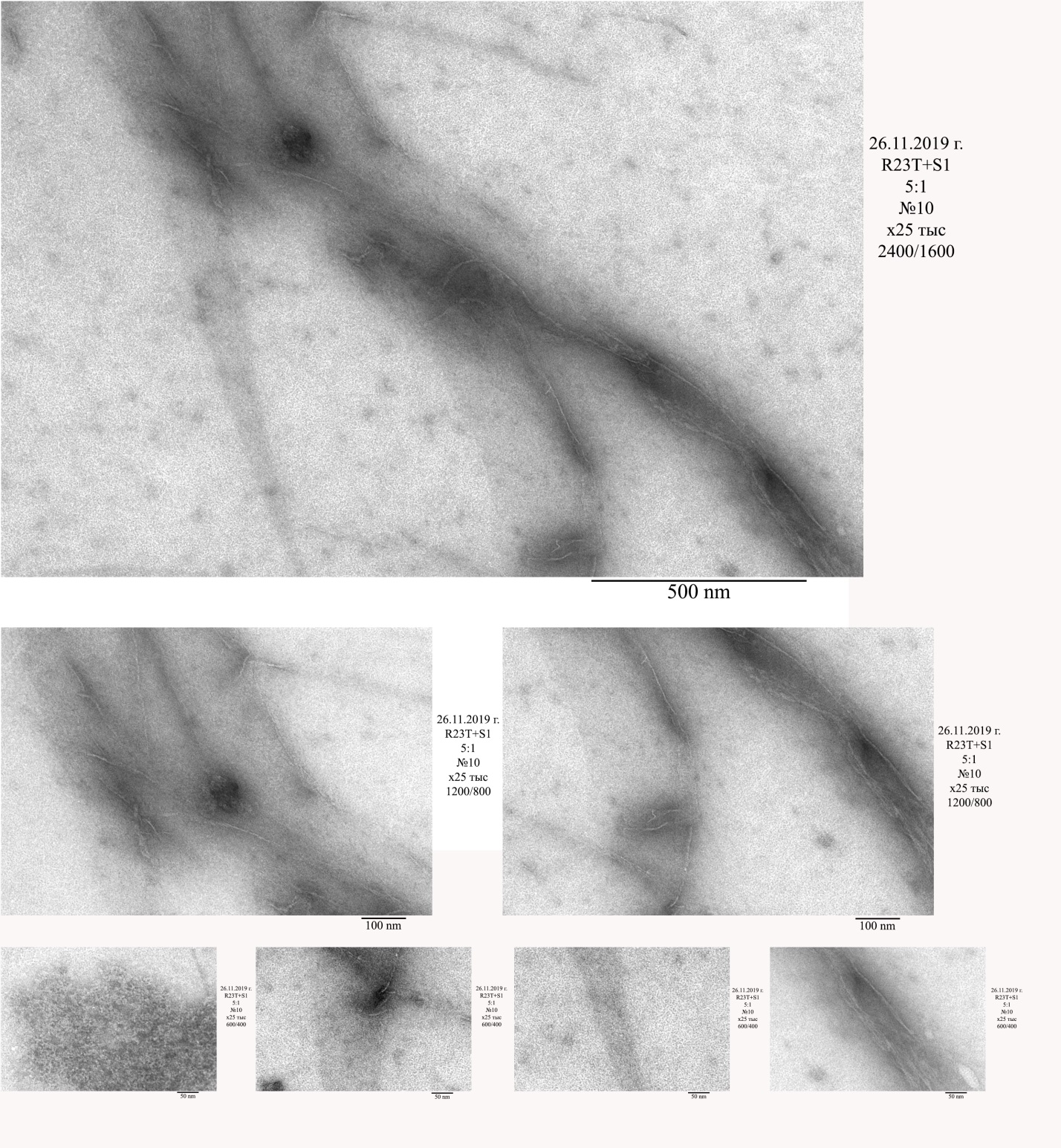 | According to the data of fluorescence spectroscopy, during the joint incubation of protein and peptide, the fluorescence intensity increases (from 3-4 to 5 units at a ratio of 1:1 and up to 11 units at a ratio of 1:5), which indicates the absence of formation of amyloids or their insignificant amount (at a ratio S1: R23T = 1:5).  When the peptide and protein are incubated together in a 5:1 ratio, both fibrils and films are formed. Films are thin, often blend into the background. One can also see aggregates that differ greatly in morphology and consistency - the constituent components of the aggregates can be small, like those of peptide aggregates, and larger. |
| 4 |  | S1:R23R=1:5 (0.2 mg/ml and 1.0 mg/ml) from *P. aeruginosa*  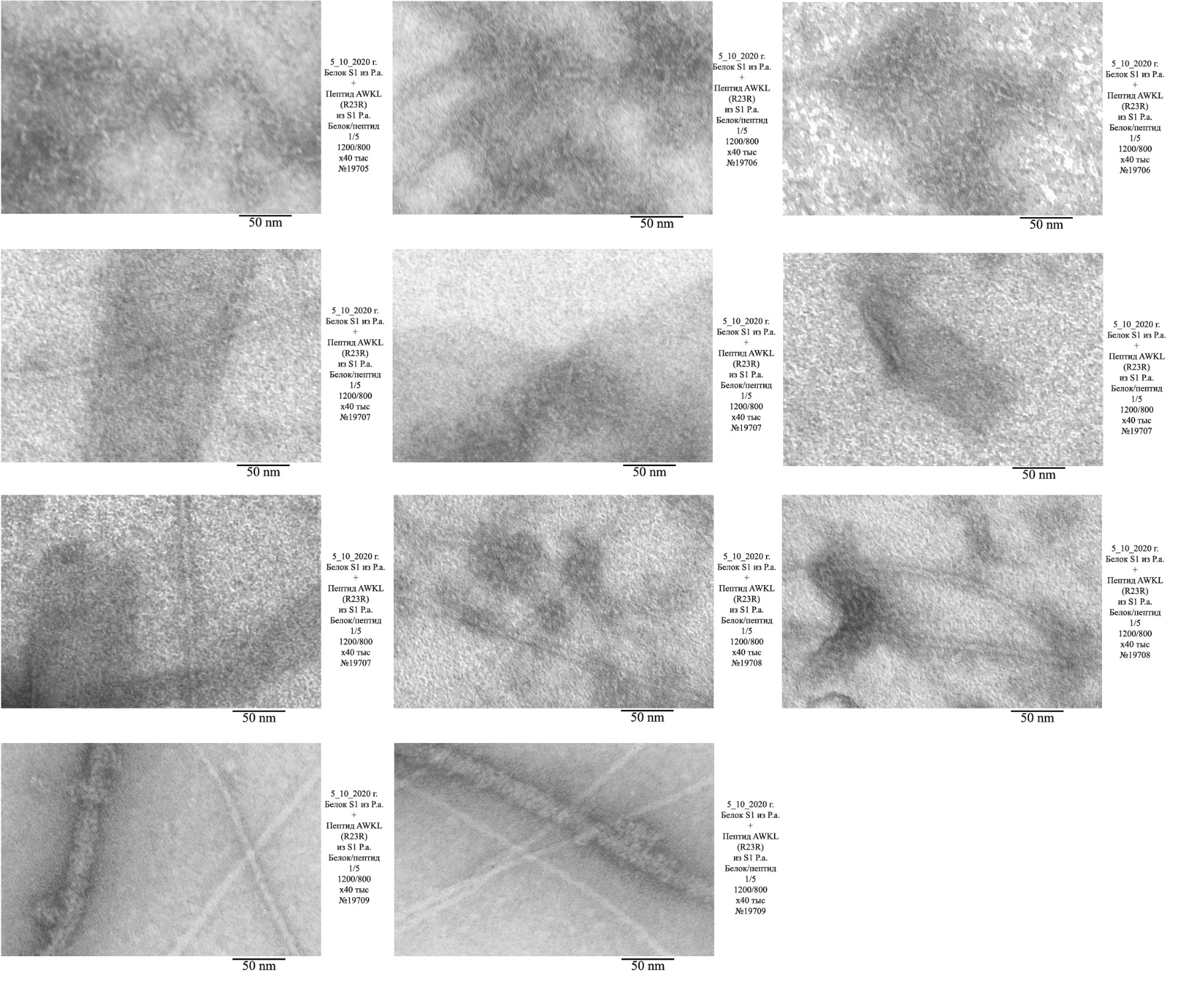 | During co-aggregation of the S1 protein and R23R from *P. aeruginosa*, both aggregates of different sizes and fibrils of different diameters are observed. In addition, film-like polymers can sometimes be observed. |
| 5 |  | S1:R23L=1:5 (0.2 mg/ml and 1.0 mg/ml) from *P. aeruginosa*  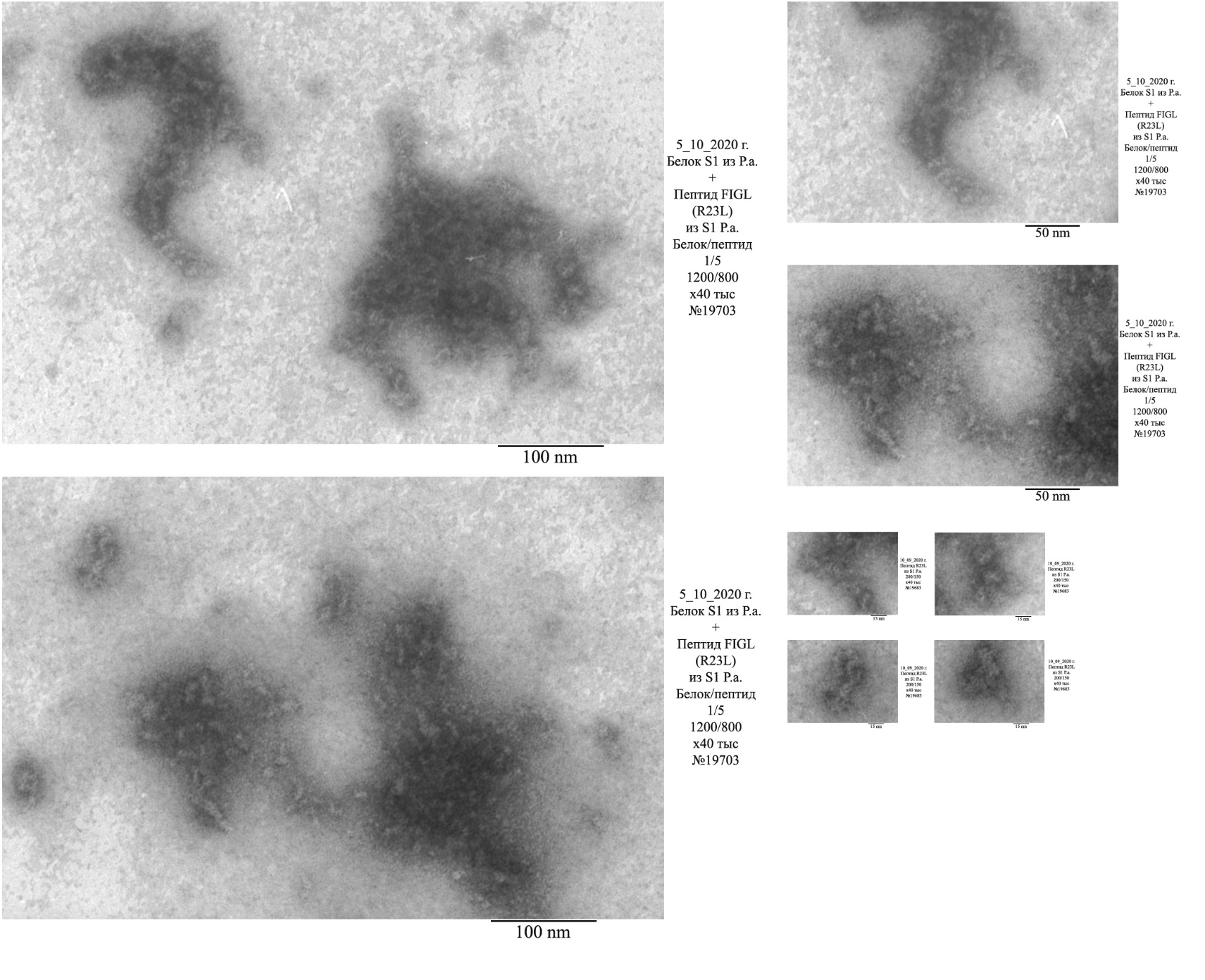 | During co-aggregation of the S1 protein and the R23L peptide from *P. aeruginosa*, aggregates of different sizes are observed. |
